# Synergy Between Low Dose Metronomic Chemotherapy and the pH-Centered Approach Against Cancer

**DOI:** 10.3390/ijms20215438

**Published:** 2019-10-31

**Authors:** Tomas Koltai, Rosa A. Cardone, Stephan J. Reshkin

**Affiliations:** 1 Via Pier Capponi 6, 50132 Florence, Italy; 2 Department of Biosciences, Biotechnology and Biopharmaceutics, University of Bari, 70126 Bari, Italy; rosaangela.cardone@uniba.it

**Keywords:** cancer reversed pH gradient, cancer proton transporters, angiogenesis, metastasis, cancer metabolism

## Abstract

Low dose metronomic chemotherapy (MC) is becoming a mainstream treatment for cancer in veterinary medicine. Its mechanism of action is anti-angiogenesis by lowering vascular endothelial growth factor (VEGF) and increasing trombospondin-1 (TSP1). It has also been adopted as a compassionate treatment in very advanced human cancer. However, one of the main limitations of this therapy is its short-term effectiveness: 6 to 12 months, after which resistance develops. pH-centered cancer treatment (pHT) has been proposed as a complementary therapy in cancer, but it has not been adopted or tested as a mainstream protocol, in spite of existing evidence of its advantages and benefits. Many of the factors directly or indirectly involved in MC and anti-angiogenic treatment resistance are appropriately antagonized by pHT. This led to the testing of an association between these two treatments. Preliminary evidence indicates that the association of MC and pHT has the ability to reduce anti-angiogenic treatment limitations and develop synergistic anti-cancer effects. This review will describe each of these treatments and will analyze the fundamentals of their synergy.

## 1. Introduction

### 1.1. Metronomic Chemotherapy (MC) vs. Maximum Tolerated Dose (MTD) Chemotherapy

Classical conventional chemotherapeutic regimens are designed to kill as many tumor cells as possible by treating them with “maximum tolerated doses” (MTDs) of cytotoxic agents. Side effects such as neurotoxicity, cardiotoxicity, and damage to proliferating cells in healthy tissues (hematopoietic system, intestinal mucosa, etc.) pose serious problems and limitations to this chemotherapy.

Metronomic chemotherapy (MC) is defined as frequent uninterrupted administration of low dose cytotoxic chemotherapy for long periods of time. The doses should be low enough to avoid myelo-suppression, but at the same time, high enough to kill endothelial cells.

Although many researchers before 1971 had described the association between growing malignant tumors and new vessels [1], it was Judah Folkman who reported for the first time that solid tumors were angiogenesis-dependent [2]. Angiogenesis means the development of new blood vessels from preexisting ones [3]. Folkman’s seminal discovery was that tumors could not grow without angiogenesis. Early in tumor development an angiogenic switch occurs activating endothelial cells in the malignant tissue. Activated endothelial cells degrade the extracellular matrix, proliferate and migrate generating new vascular sprouts [4].

He also hypothesized that there might be an unknown factor produced by the tumor that increased its vascular supply [5]: “the tumor angiogenesis factor”. Three different research groups independently and almost simultaneously (1983–1989) isolated and purified the tumor angiogenesis factor which was named Vascular Endothelial Growth Factor (VEGF) [6,7,8].

### 1.2. Anti-Angiogenic Treatment

Fumagillin, a toxic fungal product was the first anti-angiogenic drug discovered by the Folkman group, and a few years later a non-toxic derivative, TNP470, was synthesized [9]. α interferon was also successfully used for anti-angiogenic treatment of hemangiomas. Thrombospondin was identified as a natural inhibitor of angiogenesis via its inhibition of the synthesis of both VEGF [10,11,12] and FGF (fibrinogen growth factor) [13,14,15]. Angiogenesis can also be inhibited by blocking VEGF’s receptor: VEGFR [16,17,18]. From the beginning of the 1990s full-fledged research for anti-angiogenic compounds developed many new molecules like monoclonal antibodies against VEGF, 2-methoxyestradiol [19], the re-discovery of the anti-angiogenic properties of old drugs such as thalidomide [20], and low-dose chemotherapeutic agents when used metronomically, were also found to prevent angiogenesis [21].

**Figure 1:**
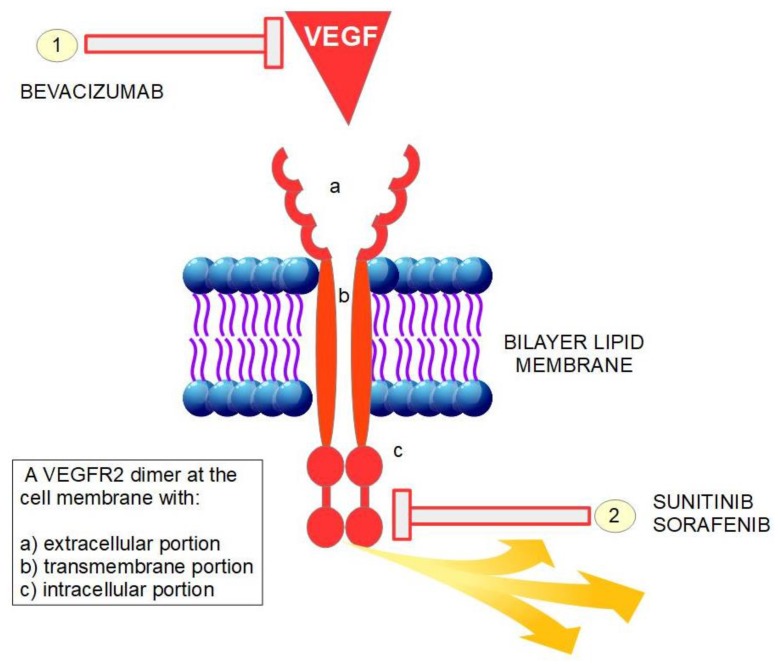
Two different mechanisms of anti-angiogenic therapy.

At the beginning of the twenty-first century two different types of anti-angiogenic drugs have represented the proof of concept of anti-angiogenesis (Figure 1):

(1) Bevacizumab: a monoclonal antibody that binds with VEGF.
(2) Sunitinib and sorafenib: small protein inhibitors of VEGF receptor tyrosine-kinases.

While the anti-angiogenic compounds have had impressive success in preclinical models, and many new agents are entering into clinical trials, this success proved short lived in bedside medicine [22,23,24]. This lack of better clinical results is the consequence of the development of resistance to anti-angiogenic treatments [25,26,27]. The resistance can be [28]:

(A) Primary: no response at all and supposedly due to intrinsic factors.
(B) Secondary or evasive: initial response and then resistance.

At the beginning of the “anti-angiogenic era” it was a widespread belief that resistance would not appear [29], because the treatment is directed against endothelial cells that are not mutated, have no genetic instability and are not supposed to over-express pro-mitotic pathways. Now we have evidence that this is not so. Tumor-associated endothelial cells (TAECs) are different from their normal tissue counterparts. Genes expressed in TAECs differ from normal endothelial cells. Croix et al. identified 79 genes that were differently expressed in TAECs [30], and Lu et al. [31] found more than 400 genes differentially expressed in ovarian cancer TAECs. Akino et al. [32] also found frequent cytogenetic changes in renal cell carcinoma TAECs. Endothelial cells of glioma are resistant to apoptosis by over-expressing GRP78 (a stress chaperone protein) [33] and survivin [34]. This creates resistance to conventional chemotherapies. Therefore, TAECs can develop resistance to treatment and this is one of the reasons for the failure of the anti-angiogenic approach.

However, we believe that the lack of the real success of anti-angiogenesis rests on a biased concept of angiogenesis, because it is much more than the VEGF-VEGFR interaction. Some of this “much more” concept is known, but there remains a large gap of unknown factors. The “much more” participants in the angiogenic process has been named the “angiome” [35,36,37] in which 1233 proteins have been identified as playing a role in the angiome. Therefore, addressing angiogenesis by downregulating or inhibiting VEGF alone seems sort of naïve. Among the known factors are other growth factors that induce angiogenesis besides VEGF:

(1) acid and basic fibroblast growth factor (FGF) [38,39]
(2) angiogenin [40]
(3) transforming growth factor α [41] and β [42,43]
(4) epidermal growth factor (EGF) [44,45], and EGF receptor (EGFR) [46,47,48];
(5) fibroblast growth factor (FGF) [49];
(6) platelet-derived growth factor [50,51,52];
(7) placental growth factor [53];
(8) hepatocyte growth factor/scatter factor [54,55];
(9) insulin-like growth factor [56];
(10) nerve growth factor [57];
(11) neuronal and glioma-derived stem cell factor [58].

This list of growth factors inducing angiogenesis explains clearly why the inhibition of VEGF or VEGFR is insufficient to fully stop angiogenesis or its recurrence. Placental growth factor is the only one that binds VEGFR, while the rest bind their own receptors. This was called “the growth factor redundancy [59]”. This angiogenic growth factor redundancy explains the limited results of targeting only one of them. Furthermore, the added hypoxia produced by anti-angiogenic treatments is an inducer for the expression of other growth factors [60] and explains the increased rate of metastasis found after anti-angiogenic treatments [61].

**Scheme 1:**
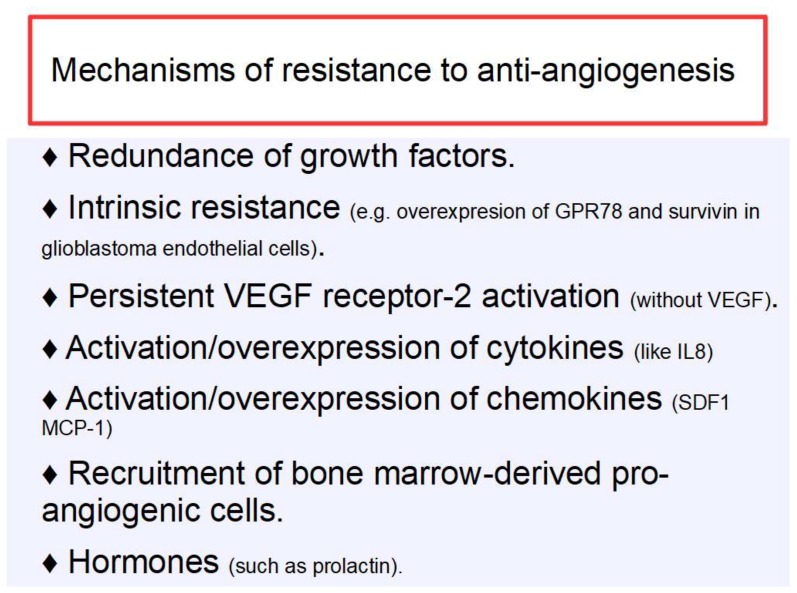
Mechanisms of resistance to anti-angiogenesis.

There are cytokines that act in a similar way to growth factors, e.g., interleukin 8 (IL 8) [62,63] ephrin A1, ephrin A2, etc. These proteins are usually over-expressed in the escape from anti-angiogenesis. Some chemokines like SDF1/CXCR4 are also angiogenesis inducers [64,65,66]. Chemokines of the CXC family containing an ELR motif are strong angiogenesis inducers while those that lack this motif show an antagonistic action [67]. Other angiogenic proteins frequently found in tumor stroma include the heparin-binding protein CYR61 that promotes migration and adhesion of endothelial cells inducing neovascularization [68], MCP-1 (monocyte chemotactic protein 1) [69], CEA related cell adhesion molecule-1 [70], tumor necrosis factor α [71], brain-derived neurotrophic factor [72], platelet-derived endothelial cell growth factor [73], BMP2 (bone morphogenetic protein 2) [74] are among other angiogenic proteins frequently found in tumor stroma. Endothelial cells over-expressing the fatty acid-binding proteins FABP4 and FABP5 (fatty acid-binding proteins) are prone to migration, proliferation and angiogenic response. While FABP4 is VEGF-dependent, FABP-5 is VEGF-independent [75]. Metabotropic glutamate receptor-1 (mGluR1) is another player in angiogenesis [76]. Its inhibition (by riluzole) significantly handicaps the process of new vessel formation [77]. Endothelin-3, a vasoactive peptide released by vascular smooth muscle cells and endothelin converting enzyme-1, a protease that activates endothelin-3 also play a role in angiogenesis [78,79,80] and especially in tumor angiogenesis [81,82,83,84,85]. See Scheme 1.

Some hormones have also been identified as pro-angiogenic. This is the case of prolactin. Prolactin is the best example of the complexity of the angiogenesis problem. While the intact prolactin molecule is angiogenic, the N-terminal portion of this hormone is anti-angiogenic [86]. Furthermore, when this N-terminal portion of prolactin binds PAI-1 (plasminogen activator inhibitor-1), another pro-angiogenic molecule, it becomes fully anti-angiogenic [87].

Galectin-1 [88] and galectin-3 [89] are other essential proteins for angiogenesis [90,91]. Their downregulation inhibits angiogenesis and decreases tumor growth [92]. Anginex [93], a designed anti-angiogenic, seems to act through the inhibition of galectin-1 [94] without relation to the VEGF-VEGFR axis. Endoglin, an endothelial co-receptor for TGF-β, also plays a role in resistance to anti-angiogenesis [95].

**Figure 2:**
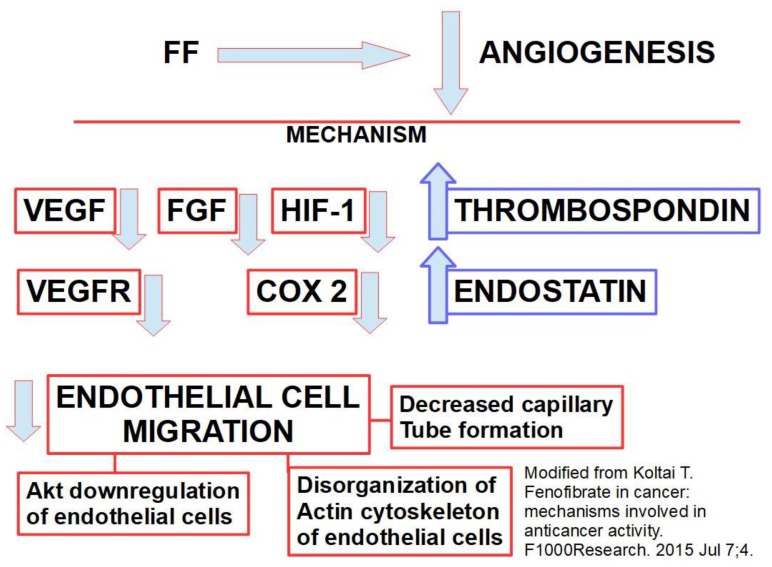
Anti-angiogenic mechanisms of Fenofibrate (FF) [113].

Using a database search to look for a complementary drug to improve and prolong the effects of anti-angiogenic treatments, we found fenofibrate (FF), a powerful anti-angiogenic drug [96] that at the same time has the ability to act on many of the angiogenic escape factors. Fenofibrate is an agonist of PPARα (peroxisome proliferator-activated receptor alpha), which is a nuclear transcription factor and is actively being used as a lipid-lowering drug. FF decreases VEGF production and increases TSP1 expression thus inhibiting angiogenesis. Added to these fundamental effects (Figure 2), FF also:

(a) Inhibits/downregulates IGF-IR [97].
(b) Inhibits the mTOR/p70S6 pathway [98].
(c) Partially inhibits the glycolytic pathway [99].
(d) Decreases TNFα and cytokines in plasma [100] and targets the TNFα/NF-kB pathway [101].
(e) Decreases the expression of HIF-1α [102,103].
(f) Inhibits 5-lipoxygenase [104].
(g) Decreases plasma platelet-activating factor (PAF) activity [105] and decreases vascular inflammatory response [106].
(h) Decreases the endothelial expression and secretion of PAI-1 (plasminogen activator inhibitor 1) [107]. PAI-1 is an indicator of poor prognosis in some cancers such as breast [108] and lung adenocarcinoma [109].
(i) Decreases EGFR activity [110,111].
(j) Increases p53 activity [112].

Treating a small number of dogs with natural cancers with the metronomic chemotherapy scheme based on cyclophosphamide, celecoxib and cimetidine a rate of response (complete response, partial response and stable disease) of 50% was achieved that increased to 75% when fenofibrate was added to the scheme (unpublished data). However, resistance to treatment appeared within twelve months.

A similar response rate and time to develop resistance was found when another group of dogs was treated with pH-centered therapies.

### 1.3. The pH Centered Therapies

Cancer cells need a very particular pH homeostasis in order to sustain growth and invasion in a very different environment from that found in normal tissues.

Normal cells have a slightly alkaline intracellular pH (pHi), around 7.1, and a much more alkaline extracellular pH (pHe), around 7.35. The difference seems very small, but pH is a logarithmic expression of H^+^ (proton) concentration. Therefore, a difference of 0.25 in pH means a great difference in proton concentration inside and outside the cell. These pH values are strictly controlled and maintained. Cells need a higher pHi in order to proliferate [114,115,116,117,118,119] and this is concordant with the fact that microtubules assembly and motility also require a higher pHi [120].

A very early step of the malignant transformation is an increase of pHi [121]. Furthermore, cancer cells require a lower extracellular pH (pHe) for motility, degradation of extracellular matrix [122], invasion [123,124] and metastasis [125,126]. Hypoxia, a major contributor to tumor development and progression [127] is a strong player in the alterations of pH in cancer. The first step in the pH modification is induced by the genetic mutation(s) that leads or lead to cancer by alkalinizing intracellular pH. Increased intracellular alkalinity, the resultant metabolic switch and hypoxia work together to drive extracellular acidification, achieving pHe values well below those of normal tissues. The metabolic switch is probably a consequence of hypoxia coupled with intracellular alkalinization. This metabolic switch consists of two features:

(a) oxidative mitochondrial metabolism is reduced while glycolytic cytoplasmic metabolism is increased;
(b) this creates a decreased production of energy which is compensated by increasing glucose uptake and metabolism.

The increased but energetically inefficient cancer cells’ metabolism increases the production of acidic molecules: lactic acid and CO_2_. The acidic load thus produced must be swiftly extruded from the malignant cells in order to maintain an alkaline cytoplasm that favors proliferation. The consequence of this acid extrusion is microenvironmental acidification. At this stage, the intracellular pH is higher than the pH of the microenvironment: inversion of the pH gradient.

Now, the pH frame is perfect for increased proliferation (increased pHi) and mobility, migration, invasion, degradation of the extracellular matrix, angiogenesis, and metastasis (decreased pHe). As an added value, extracellular acidity also permits the escape from the immune system and resistance to drug therapy. The group headed by Barber called this dysregulated pH situation “the perfect storm” [128].

There are multiple cellular mechanisms involved in creating the inversion of the pH gradient and keeping it that way throughout the cancer’s life. The main mechanisms are represented by a wide range of different membrane-associated proteins working as channels, exchangers, transporters or enzymes. We have called all the elements participating in the creation of the perfect storm the “pHtome”, namely:

(1) Na^+^/H^+^ exchangers (NHEs) and specifically the NHE1 isoform: promote the reversible electroneutral exchange of Na^+^/H^+^. In cancer, these exchangers extrude H^+^ from the cytoplasm to the extracellular space.
(2) Monocarboxylate transporters (MCTs) and specifically the MCT1 and 4 isoforms. These MCTs extrude lactate and proton associated with lactate. The metabolic switch that increases glycolytic metabolism and decreases oxidative phosphorylation overloads the cell with lactate which is removed from the cell through MCTs.
(3) Membrane carbonic anhydrases (CAs) isoforms 9 and 12 (CAIX and CAXII) mediate the reversible hydration of CO_2_ (which is produced in excess in malignant cells) producing carbonic acid.
(4) V-ATPase proton pumps extrude protons across membranes while consuming energy.
(5) Anion Exchangers (AEs) mediate the electroneutral transmembrane exchange of HCO3^-^ by Cl^−^.
(6) Specificity protein 1 (Sp1) is a transcription factor and enhancer that increases the transcription of HIF-1α, CAIX, NHE1, and some protein domains that form the V-ATPase proton pump.
(7) Voltage-gated sodium channels (VGSCs) are channels that upon a stimulus incorporate Na^+^ into the cell. While intracellular Na^+^ has a role in increasing pHi, the main action of VGSCs in the pH inversion scheme are related to NHE1 activation.

The inversion of the pH gradient in cancer is a constant finding and there is compelling evidence that more than one of these pH regulators are the engines driving the inversion.

The fundamentals behind the pH-centered treatment of cancer are based on the following concepts:

(1) The inversion of the pH gradient (the perfect storm) is not merely an “innocent” consequence of cancer progression, but an important etiopathogenic and determinant factor in the origin and development of cancer and its progression.
(2) The inverted pH gradient is a constant finding in all types of malignant tumors.
(3) The proteins involved in this process are the components of the pHtome.
(4) Reverting the inverted pH gradient creates an inadequate environment for cancer growth and progression that leads to apoptosis or at least to a slowing down of proliferation and invasion.
(5) This means that the proteins of the pHtome must be downregulated, removed, blocked or inhibited.
(6) It is useless to inhibit only one of the soldiers, because the others would take up the functions of the lost comrade.
(7) It is not possible to fully systemically attack the proteins, because most of them also perform other functions that are beneficial and necessary for normal cells (housekeeping proteins).
(8) However, it is possible to downregulate or decrease the activity of many of them without affecting normal cells.
(9) The simultaneous and partial inhibition of many of the participants of the pHtome will decrease tumor progression.
(10) The partial inhibition of the pHtome does not only go along with other chemotherapeutical approaches, but also improves their results.

A treatment based on these concepts uses:

(a) an NHE1 inhibitor such as amiloride, a diuretic that has been used for almost fifty years;
(b) an inhibitor of carbonic anhydrases such as acetazolamide, another diuretic which has been in clinical practice since the 1940s;
(c) a proton V-ATPase inhibitor such as esomeprazole, lansoprazole, pantoprazole all of which are being used for the treatment of acid-related diseases of the upper gastrointestinal tract [129,130,131];
(d) a MCT inhibitor like the nutraceutical quercetin wrongly considered to be a food supplement that is sold over-the-counter, but which has clear pharmacological effects;
(e) a VGSC inhibitor like topiramate, used as an anticonvulsant in the treatment of epilepsy; but which is also a CA inhibitor.

Unfortunately, there are no known AE inhibitors that can be used in clinical practice; those that are available are exclusively suitable for experimental purposes and are toxic enough to preclude them for in vivo experiments.

The five types of drugs listed above represent the core of the pH-centered treatment.

When a small group of dogs with natural cancers was treated with both schemes (MT and pHT) the overall survival increased from 9–11 months to an average of 22 months in those who responded to treatment. (See Supplementary Table). It is premature to draw conclusions from this finding because the different groups of dogs considered in this review were quite different regarding breed, age, previous treatments, and types of cancers. However, the difference in overall survival is significant enough to encourage testing the MT-pHT association in a standardized larger trial.

**Figure 3:**
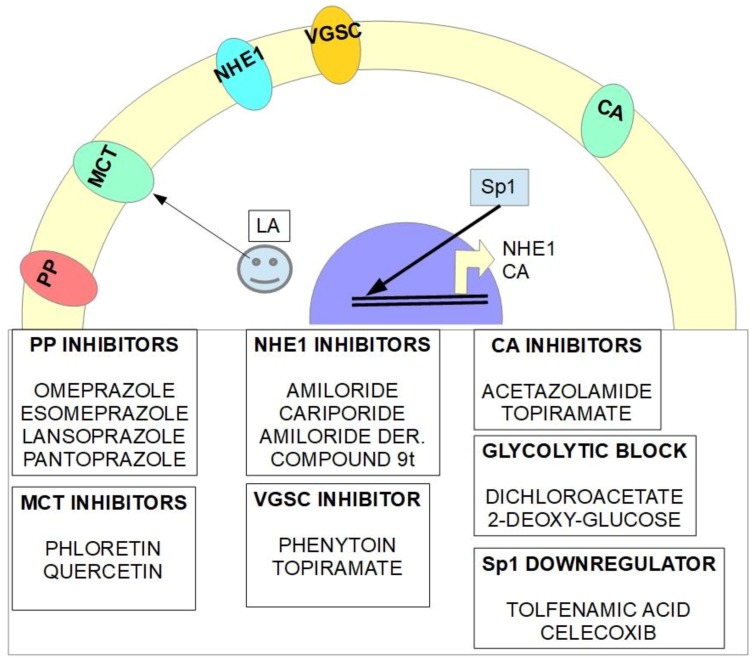
The combination of different proton-extruder inhibitors (“a cocktail”) are the base of the pH centered treatment of cancer with two goals: Acidifying the cytoplasm and alkalinizing the extracellular pH.

On a theoretical basis there are many issues that can explain the apparent synergy between both treatments:

❖ Acetazolamide decreases angiogenesis probably by inhibiting aquaporin 1 [132,133,134].
❖ Topiramate has anti-angiogenic effects [135,136] and has shown dose-dependent VEGF downregulation in some types of cancers [137].
❖ Proton pump inhibitors induce endothelial cell senescence [138]: Chronic use of PPIs impaired endothelial function through telomere length reduction.
❖ Amiloride decreases angiogenesis [139,140,141,142,143].
❖ Quercetin is also an anti-angiogenic drug [144,145,146,147,148,149].
❖ NSAIDs such as tolfenamic acid or celecoxib downregulate Sp1, which is a transcription factor for HIF-1α, CAIX, V-ATPase proton pumps and NHE1. Downregulation of HIF-1α decreases expression of VEGF, VEGFR1, and VEGFR2 [150] (Figure 3).

From the extensive evidence showed above, it is clear that the pHT has an anti-angiogenic effect. However, this does not explain why the MC-pHT association effects extend for a longer period than when they are used separately.

The first reason is probably based on the shared characteristic of the pHT drugs: they all increase extracellular pH (and at the same time decrease intracellular pH).

The next question is: what is the relationship between extracellular acidity and anti-angiogenic treatment?

Faes et al. [151] found that sorafenib (a VEGFR tyrosin-kinase inhibitor) combined with sodium bicarbonate had a stronger anti-tumoral effect than each drug used independently. They also found that the reason behind this was a higher number of endothelial cells expressing VEGFR as pHe increased and suggested that strategies that increase pHe improve anti-angiogenic treatment outcomes. This would also explain the better results found with the MC-pHT association.

Shi et al. [152] found that acidosis increased VEGF expression. Both publications, (Faes et al., and Shi et al.), concurred that the alkalinization of the extracellular space was adequate to decrease angiogenesis and improve the results of anti-angiogenic treatments.

### 1.4. Many Ion And Water Channels/Exchangers Downregulated by pHT Are Angiogenic

- NHE1 the pro-angiogenic exchanger is the main culprit in the pH gradient inversion. It is inhibited by pharmaceuticals such as amiloride, cariporide and derivatives [153,154,155].
- Aquaporin 1, a water channel that is inhibited by acetazolamide and topiramate, is strongly expressed in endothelial cells [156]. Aquaporin 1 plays an important role in endothelial cell migration and favors angiogenesis. In aquaporin 1 null mice the migration of endothelial cells is compromised [157]. Downregulation of aquaporin 1 decreases angiogenesis [158].
- VGSCs are pro-angiogenic. NaV1.5 and NaV1.7 are the predominant isoforms found in endothelial cells. VGSCs showed a modulatory effect on the pro-angiogenic properties of VEGF [159].
- CAIX seems to be pro-angiogenic [160]. We use the term “seems” because CAIX is a hallmark of hypoxia and increased HIF-1α activity. Therefore, it is difficult to establish whether CAIX is angiogenic *per se* or actually the hypoxia-HIF-1α-VEGF pathway is the reason for this pro-angiogenic effect. What we do know is that inhibition of CAIX enhances anti-angiogenic treatment results [161].
- V-ATPase proton pump inhibitors handicap endothelial cell proliferation and migration with inhibition of VEGFR2 signaling and decreasing the amount of VEGFR2 at the cell surface [162]. Therefore, V-ATPase proton pump inhibitors are clearly anti-angiogenic. There is laboratory and clinical evidence on using proton pump inhibitors in cancer treatment [163,164,165,166,167]. Furthermore, there is evidence showing synergy between MC and proton pump inhibition [168].

## 2. Discussion

The short benefit of clinical anti-angiogenesis is an important limiting factor of these types of therapies. Acting only on the VEGF-VEGFR axis is insufficient for achieving lasting results because many escape doors remain open [169,170,171,172]. Initial successful results are lost as soon as these escape doors open. As Quesada et al. [173] clearly stated; playing only one instrument may not be enough in anti-angiogenesis. Resistance to anti-angiogenesis is not related to mutations or to multidrug resistance mechanisms. It is related to alternative angiogenic pathways [174]. These alternative pathways are targeted by the pHT approach.

There are a few pharmaceuticals that can be added to mainstream anti-angiogenesis, such as fenofibrate or riluzole. These drugs could impede the opening of some escape routes. However, we believe that the main cooperation for lasting anti-angiogenesis would come from alkalinizing the extracellular space, which is highly acidic in all malignancies. The best way to achieve extracellular alkalinization is through intracellular acidification. The benefits of extracellular buffers like sodium bicarbonate are short lived, while the partial inhibition of many participants of the pHtome, like proton extruders, is well tolerated by patients and has more lasting effects.

The incubation of human pancreatic cancer cells in an acidic medium increased the expression of IL-8. When cells with low IL-8 expression were implanted in the pancreases of mice they showed lower angiogenesis than those with higher IL-8 expression [175]. IL-8 is a pro-angiogenic cytokine and serves as an example of low pHe promoting angiogenesis.

Solving the acidity problem of the extracellular space with a pH-centered approach should have two different but converging effects on angiogenesis.

### 2.1. It Would Decrease the VEGF-VEGFR Axis Activity

**Figure 4:**
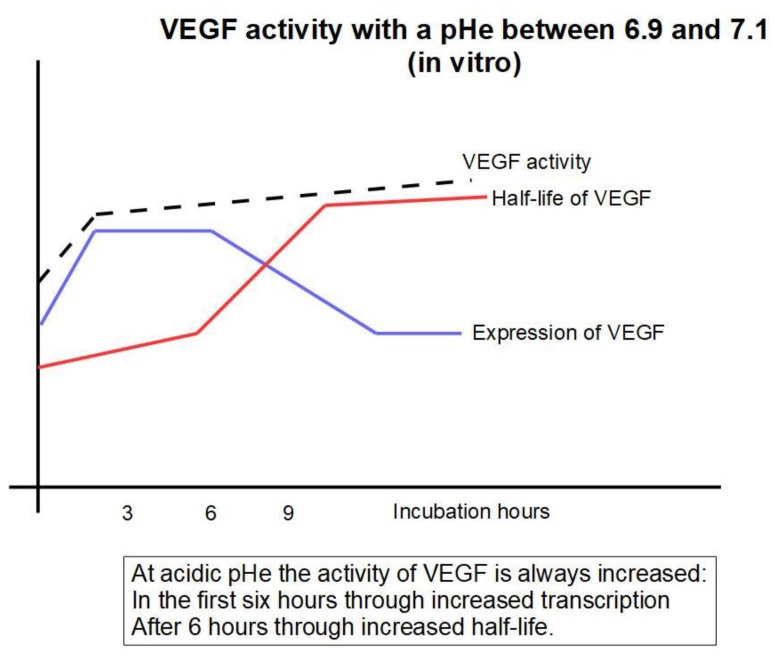
Explanation in the text.

However, this might seem a controversial issue because VEGF expression does not show a linear correlation with extracellular acidity. When a malignant cell was placed in a low pH medium, VEGF expression was increased during the first 6 h of incubation. Further incubation showed increased VEGF expression at pH 6.9 to 7.1 but a decrease at longer incubation times [146]. This, at *prima facie*, seems to be the opposite of what would be expected in cancer. In a second step of their investigation, the authors found that “the VEGF mRNA half-life of cells at a pH of 6.9 was much longer than that of cells at pH 7.4”. Thus, the determination of VEGF expression does not reflect what happens with VEGF’s activity that is increased at low extracellular pH. In other words, after the first six hours in which low pHe increased the synthesis of VGEF at transcriptional level, the low pHe increased the activity of VEGF at a post-transcriptional level. Raising extracellular pH level decreases VEGF activity at the transcriptional level first and at the post-transcriptional level afterwards (Figure 4).

### 2.2. It Would Decrease the Activity/Expression of Other Molecules Involved in Angiogenesis And in Anti-Angiogenic Escape

We have already mentioned the increased IL-8 expression with low pHe. The TRPV4 (transient receptor potential vanilloid 4; also known as vanilloid receptor) ion channel is another player in angiogenesis [176,177,178,179] that is activated by low pHe [180,181]. Acidosis also induces the expression and modulates the activity of SDF-1α in both normal [182,183] and in malignant cells [184]. Acidic preconditioning improves endothelial cells colony formation and angiogenesis [185]. Furthermore, Galectin-3 is up-regulated by acidosis [186].

The pHT approach decreases cellular migration by multiple mechanisms:

(a) It increases extracellular pH, which decreases the activity of proteolytic cathepsins, necessary for matrix degradation.
(b) It decreases in the same way metalloproteases maturation also necessary for matrix degradation.
(c) It decreases invadopodia formation and activity.
(d) It inhibits aquaporin 1 which is essential for migration.

These activities are not restricted to malignant cells but also affect tumor-associated endothelial cells, therefore pHT has a clear anti-angiogenic effect that acts in a different way than the VEGF-VEGFR pathway inhibition.

At high doses amiloride derivatives induce endothelial cell apoptosis in extracellular alkalosis [187]. Amiloride and its derivatives also inhibit endothelial production of MCP1 (monocyte chemoattractant protein 1) [188] that is a pro-angiogenic protein [189,190]

From the above evidence, we can see that pHT addresses many issues that are “left out” from classical anti-angiogenic treatments. The synergy between the two approaches is explained by the fact that pHT complements the anti-angiogenic treatments.

A third pillar has been proposed for this combined MC-pHT approach: targeting the immune system dysfunction. McDonald et al. [191] suggest the use of immune checkpoint inhibitors. These modern drugs that have been shown to be very useful in the treatment of many types of cancer are however very expensive and produce many side effects that are not tolerated by all patients.

The advantage of metronomic chemotherapy based on cyclophosphamide and cimetidine is that it is quite efficient in targeting immune dysfunction without all the side effects of immune checkpoint inhibitors and its exorbitant cost.

In this respect, Loges et al. [192] wrote about the need to develop third generation anti-angiogenic drugs taking into account the resistance problem. We consider that the pHT-MC association somehow, represents a third generation scheme of anti-angiogenesis.

Studies supporting this concept are shown in Table 1 and Table 2.

Conclusions from Table 1: Low dose cyclophosphamide and low dose metronomic cyclophosphamide (50 mg per os, daily, with no interruptions) not only have anti-angiogenic effects but also enhance immunologic modulation against cancer by decreasing T regulatory cells and increasing T-helper lymphocytes and NK cells.

For detailed recent reviews on cyclophosphamide as an immune regulator in cancer, read Hughes et al. [202] and the phase I clinical trial of metronomic cyclophosphamide and everolimus by Huijts et al. [203].

Conclusions from Table 2: Cimetidine antagonizes histamine’s immuno-suppressive effects and has powerful stimulatory effects on blood cells such as neutrophils and monocytes, and also macrophages, dendritic cells, NK cells, T-helpers, and CD8^+^ cytotoxic T cells [214]. For a review of cimetidine’s immunological actions see Pantziarka et al. [215]. The usual dose of cimetidine is 400 mg three times a day.

The cimetidine-cyclophosphamide association has synergistic antitumoral effects [216,217].

While this association in a metronomic scheme restores immunological defenses and has anti-angiogenic activity, bevacizumab has not shown immuno-modulatory abilities. Therefore, the metronomic low dose cyclophosphamide plus cimetidine restores immunologic functions that makes immune checkpoint inhibitors unnecessary in a scheme of MC plus pHT.

## 3. Conclusions

Tumor angiogenesis is much more complex than originally thought. Although VEGF and VEGFR seem to be the main players, the limited bedside success achieved with their inhibition shows that there are many other players that must be targeted. Present day treatments do not address all the other participants in the process. The direct result of this biased view of anti-angiogenesis is resistance to treatment. On the other hand, resolving the pH paradigm of cancer seems to delay this phenomenon substantially. pH-centered treatment targeting channels, transporters, exchangers, and enzymes that form part of the pHtome seems to have an impact on anti-angiogenesis. Evidence, that still needs further confirmation, shows that pH centered treatments would be a good companion to classic anti-angiogenesis therapies. Riluzole, a potent inhibitor of the pro-angiogenic protein kinase C and antagonist of the also pro-angiogenic metabotronic glutamate receptor-1 on one side and voltage gated sodium channel inhibitors on the other, should also be incorporated into anti-angiogenic and pH centered treatments because this adds other three targets to the anti-angiogenic scheme. Alkalinization of the extracellular space that is achievable through pHT is a powerful enhancer of anti-angiogenic treatments and delays resistance.

## Figures and Tables

**Table 1:** Immune effects of low dose cyclophosphamide (LDC) and low dose metronomic cyclophosphamide (LDMC).

Reference	Findings
Berd et al. 1988 [193]	LDC showed progressive decrease of T suppressor CD4^+^ expressing 2H4^+^ cells. True helper cells were not reduced.
Ghiringhelli et al. 2004 [194]	A single dose of LDC depleted CD4^+^CD25^+^ T regulatory cells in tumor bearing animals, and significantly increased the effects of subsequent immunotherapy.
Lutsiak et al. 2005 [195]	LDC inhibited T cell regulatory cells CD4^+^CD25^+^ not only decreasing their number but also their functionality.
Ghiringhelli et al. 2007 [196]	In very advanced cancer patients, LDMC downregulated CD4^+^CD25^+^ regulatory T cells and at the same time restored the functionality of T and NK cells.
Brode et al. 2008 [197]	LDC inhibited regulatory T cells through inhibition of Foxp3^+^.
Xhao et al. 2010 [198]	LDC produced selective depletion of CD4^+^CD25^+^ Foxp3 Tregs by depleting intracellular ATP.
Burton et al. 2011 [199]	11 dogs with soft tissue sarcoma treated with LDMC showed decreased Tregs and decreased angiogenesis
Ge et al. 2012 [200]	For 3 months the authors studied the effects of LDMC in 12 patients with treatment-refractory breast cancer with metastases. Initially there was a significant decrease in circulating Tregs for a short period. Furthermore, there was an important increase in reactive T cells that remained at high levels during the 3 months of the study. This meant that the recuperated activity of reactive T cells outlasted the short lived Treg decrease.
Wu et al. 2015 [201]	Mice with implanted glioma treated with LDMC showed an increase in tumor associated-cytotoxic CD8^+^ lymphocytes, NK cells and macrophages achieving tumor regression.

**Table 2:** Cimetidine’s immunologic effects.

Reference	Findings
Osband et al. 1981 [204]	Cimetidine produced inactivation of suppressor cells, slowed metastases development and prolonged survival in tumor bearing mice.
Ershler et al. 1983 [205]	Mice treated with daily injections of cimetidine produced twice as much specific antibody in response to immunization.
Jin et al. 1986 [206]	Cimetidine induced loss of suppressor cells function.
Hirai et al. 1987 [207]	Cimetidine increased the interferon α enhancer effect on NK cell activity. Cimetidine decreased interferon α production, therefore both drugs have to be administered together in order to increase NK’s activity.
Sahasrabudhe et al. 1987 [208]	Cimetidine decreased suppressor T lymphocytes and decreased their activity.
Dejiao et al. 1999 [209]	Peri-operative administration of cimetidine in patients with colon cancer significantly increased NK cells in the surgical specimen as compared with patients not receiving it.
Kubota et al. 2002 [210]	Cimetidine increased the antigen presenting capacity of dendritic cells in tumors obtained from patients with colorectal cancer.
Zheng et al. 2013 [211]	Cimetidine induced apoptosis in myeloid derived suppressor cells inhibiting lung tumor growth in mice in vivo. It also increased interferon γ production.
Pan et al. 2015 [212]	Cimetidine inhibited T-cell suppression by myeloid derived suppressor cells.
Vila-Leahey et al. 2016 [213]	Ranitidine, another histamine receptor 2 antagonist, also significantly decreased the population of myeloid derived suppressor cells in spleen and bone marrow and decreased lung metastasis in tumor xenografted mice.

## Supplementary Materials

Supplementary materials can be found at https://www.mdpi.com/1422-0067/20/21/5438/s1.
