# Supplementary material for: Synergy Between Low Dose Metronomic Chemotherapy and the pH-Centered Approach Against Cancer
Source: Int J Mol Sci. 2019 Oct 31;20(21):5438. doi: 10.3390/ijms20215438 (PMC6862380; doi:10.3390/ijms20215438)
Supplement: Supplementary file 1 [file ijms-20-05438-s001.pdf]

Supplementary Table

All the dogs shown in the Table were treated with informed consent of their owners.

Dog	Diagnosis	Chemotherapy	Surgery
	Treated with Metronomic Chemotherapy		
1	Sertoli testicular tumor with lung metastasis	No	No
2	Non removable squamous cell carcinoma of gum	No	No
3	High grade mast cell tumor with liver and spleen metastasis	No	No
4	Femoral osteosarcoma	?	Yes
5	Tubulopapillary carcinoma of mammary gland with metastases.	No response	Yes
6	Anaplastic carcinoma of mammary gland with metastasis	No response	Yes
7	Multicentric diffuse lymphoblastic lymphoma.	No response	No
	TREATED WITH pH CENTERED APPROACH		
1	Gastric adenocarcinoma	No	No. Poor risk.
2	Hypodermal hemangiosarcoma with metastases	No	Yes
3	Oral melanoma with lymph node metastases	No	No
4	Oral melanoma with lung metastases	No	No
5	Hepatic Leomyosarcoma with metastases	Yes. No response	Yes
6	Rhabdomyosarcoma of leg with metastases	No	Yes
7	Squamous cell carcinoma of one tonsil with node metastases	Yes. No response	No.Big bulk.
	TREATED WITH BOTH THERAPIES		
1	Relapsing abdominal cavity liposarcoma	No	Yes
2	Relapsing osteosarcoma of the mandible	No	Yes
3	Nasal carcinoma invading neighboring tissues	Yes. No response	No
4	Nasal chondrosarcoma of big size and local invasion	No	No
5	Relapsing invasive transitional cell carcinoma of bladder	Yes. No response	Yes
6	Abdominal mesothelioma	No	Yes
7	Metastatic bone cancer. No primary tumor identified	Yes. No response	No
